# Unveiling Chloroplast RNA Editing Events Using Next Generation Small RNA Sequencing Data

**DOI:** 10.3389/fpls.2017.01686

**Published:** 2017-09-29

**Authors:** Nureyev F. Rodrigues, Ana P. Christoff, Guilherme C. da Fonseca, Franceli R. Kulcheski, Rogerio Margis

**Affiliations:** ^1^Programa de Posgraduação em Genética e Biologia Molecular, Departamento de Genética, Universidade Federal do Rio Grande do Sul, Porto Alegre, Brazil; ^2^Programa de Posgraduação em Biologia Celular e Molecular, Centro de Biotecnologia, Universidade Federal do Rio Grande do Sul, Porto Alegre, Brazil; ^3^Programa de Pósgraduação em Biologia Celular e do Desenvolvimento, Departamento de Biologia Celular, Genética e Embriologia, Universidade Federal de Santa Catarina, Florianópolis, Brazil; ^4^Departamento de Biofísica, Universidade Federal do Rio Grande do Sul, Porto Alegre, Brazil

**Keywords:** small RNA, chloroplast, RNA editing, NGS, SNP genotyping

## Abstract

Organellar RNA editing involves the modification of nucleotide sequences to maintain conserved protein functions, mainly by reverting non-neutral codon mutations. The loss of plastid editing events, resulting from mutations in RNA editing factors or through stress interference, leads to developmental, physiological and photosynthetic alterations. Recently, next generation sequencing technology has generated the massive discovery of sRNA sequences and expanded the number of sRNA data. Here, we present a method to screen chloroplast RNA editing using public sRNA libraries from Arabidopsis, soybean and rice. We mapped the sRNAs against the nuclear, mitochondrial and plastid genomes to confirm predicted cytosine to uracil (C-to-U) editing events and identify new editing sites in plastids. Among the predicted editing sites, 40.57, 34.78, and 25.31% were confirmed using sRNAs from Arabidopsis, soybean and rice, respectively. SNP analysis revealed 58.2, 43.9, and 37.5% new C-to-U changes in the respective species and identified known and new putative adenosine to inosine (A-to-I) RNA editing in tRNAs. The present method and data reveal the potential of sRNA as a reliable source to identify new and confirm known editing sites.

## Introduction

Chloroplasts are notable examples of successful endosymbiosis in the early origin of modern life forms. These organelles possess their own gene expression machinery, with complex posttranscriptional processes and fine nucleus-cytosol crosstalk. In plants, these organelles undergo a posttranscriptional process called RNA editing, corresponding to nucleotide changes from cytosine to uracil (C-to-U) and less frequently from uracil to cytosine (U-to-C), in some sites of coding sequences (Tillich et al., 2006; Chateigner-Boutin and Small, 2010). These nucleotide changes correct the codons to encode appropriate amino acids, maintaining the functional amino acid sequence of the evolutionarily conserved protein (Takenaka et al., 2013). Another well-known mechanism of RNA editing is the adenine to inosine (A-to-I) editing, as observed in the chloroplast tRNA^Arg^ (ACG). This type of editing enables hydrogen bond formation with more than one base in the corresponding codon position (Su and Randau, 2011). The A-to-I editing in position 34 of the tRNA^Arg^ (ACG) produces the wobble nucleotide described as essential for efficient chloroplast translation (Delannoy et al., 2009). In *Arabidopsis thaliana*, arginine tRNA adenosine deaminase (TAD or ADAT) performs this deamination (Elias and Huang, 2005; Delannoy et al., 2009).

RNA editing in coding sequences increases the conservation levels among proteins across several plants species. Evolutionarily, codons generated by RNA editing are more conserved than codons encoded by genomic DNA (Guo et al., 2015). Editing sites located within coding sequences have been well studied, despite the existence of editing sites in non-coding regions, such as introns and tRNAs. There are several cases of different editing efficiencies from plant to plant, and even among different plant tissues (Peeters and Hanson, 2002; Chateigner-Boutin and Hanson, 2003; Tseng et al., 2013), suggesting that several different RNA editing sites remain to be elucidated.

The identification of all components from the RNA editing machinery has not yet been achieved, although several proteins have been identified as important for the maintenance of editing processes. The pentatricopeptide repeat proteins (PPR) are a highly diverse protein family. In the plant evolutionary landscape of PPR proteins, 109 genomes/proteomes were analyzed, resulting in a total of 49,204 PPR genes and 616,206 motifs (Cheng et al., 2016). Some of these PPRs harbor a DYW motif, similar to the deaminase motifs observed in other proteins, which could explain the C-to-U nucleotide conversion (Salone et al., 2007; Schallenberg-Rüdinger et al., 2013; Hayes et al., 2015). In addition, several studies have reported PPRs associated with specific RNA editing events, demonstrating that these molecules bind to specific *cis*-elements located upstream of the RNA editing site (Okuda et al., 2006; Barkan and Small, 2014). Moreover, the PPR alone is not sufficient to promote RNA editing but requires other proteins, such as RNA editing-interacting (RIP/MORF), OMMR and OZ proteins, to achieve a successful editing event (Bentolila et al., 2013; Sun et al., 2016).

The most frequent plastid RNA editing type in flowering plants is the C-to-U change, with approximately 40 sites detected thus far in Arabidopsis (Takenaka et al., 2013). To facilitate RNA editing site prediction in organelles, software, such as PREP suite has been developed (Mower, 2009). These programs enable RNA editing site prediction in genes from organelles by considering homology and conservation among protein sequences compared to genomic databases. Currently, thousands of partial and complete plastid genomes are available in NCBI, which can be used to extensively search for RNA editing events.

Different experimental techniques have identified chloroplast RNA editing sites. A widely used method is the reverse transcription PCR (RT-PCR) of plastid messenger RNAs in which several chloroplast cDNA fragments are cloned into vectors and further sequenced (Rüdinger et al., 2009). Additionally, if a chloroplast candidate gene sequence is previously known, then specific primers can be designed to direct the gene amplification from cDNA samples, with subsequent sequencing (Wolf et al., 2004). RNA editing events can also be detected through the Poisoned Primer Extension method or High Resolution Melting (HRM) analysis (Chateigner-Boutin and Small, 2007), using chloroplast cDNA as a template for amplification. Another method to measure RNA editing is multiplex RT-PCR mass spectrometry, described as a robust and convenient method (Germain et al., 2015). Although robust, these methods are dependent on specific primers and are restricted to RNA editing studies only.

RNA sequencing has facilitated RNA editing analyses by comparing reads from RNA-seq data with organelle genome references. Currently, RNA-seq is primarily adapted to study polyadenylated transcripts. Thus, as their cyanobacterial ancestor, several plastid polyadenylated RNA transcripts are associated with the RNA decay pathway *via* degradation by 3′– 5′ exoribonucleases (Komine et al., 2002; Zimmer et al., 2009). Therefore, this approach generates RNA-seq libraries with smaller amounts of plastid reads than libraries generated from organelle-enriched RNA samples, with posterior reduction of ribosomal RNA (Guo et al., 2015). Furthermore, these approaches restrict the analysis to only transcripts located in chloroplasts, preventing a comparative analysis between nuclear and plastid transcripts.

In recent years, studies of small RNAs (sRNA) have considerably increased, particularly associated with the deep sequencing of microRNAs (miRNAs) and other small non-coding RNAs (ncRNAs) from nuclear origin, producing a large amount of new sequence data. These studies have focused on the roles of sRNAs in genome maintenance, development and plant responses to environmental stresses (Simon et al., 2009; Long et al., 2015; Xu et al., 2015). However, plastid-derived sRNA sequences have also been identified in these total sRNA libraries (Ruwe and Schmitz-Linneweber, 2012; Zhelyazkova et al., 2012; Ruwe et al., 2016). Therefore, considerable amounts of sRNA data are available in public databases and can be employed for RNA editing studies. In the present study, we propose that sRNA sequencing data could represent an additional resource to identify chloroplast RNA editing events, in addition to other approaches, such as strand-specific RNA sequencing and Single Nucleotide Polymorphism (SNP). Here, we describe a method for identifying a set of new editing sites in chloroplast transcripts using sRNA data. Analyses of sRNA libraries can provide a strong qualitative and reliable quantitative measure of plastid RNA editing events.

## Materials and methods

### sRNA libraries and chloroplast genomes

Public RNA libraries deposited in NCBI GEO (www.ncbi.nlm.nih.gov/geo/) with accession numbers GSE85070 (Wu et al., 2016) (*Arabidopsis thaliana*, mRNA-seq and sRNA-seq), GSE69571 (da Fonseca et al., 2016) (*Glycine max*, soybean, mRNA-seq and sRNA-seq) and GSE77046 (Neto et al., 2015) (*Oryza sativa* japonica group, rice, sRNA-seq; mRNA-seq data unpublished) were used as input data to evaluate the proposed method. These libraries were produced from samples with no qualitative influence on RNA editing and did not use any method to enrich the isolation of plastid RNAs. The Arabidopsis mutant data present in the libraries were not used. For sRNA analyses, only reads with 18–24 nucleotides were selected from the libraries. Complete chloroplast genome, coding sequences and tRNAs from Arabidopsis (NC_000932), soybean (NC_007942), and rice (NC_001320) were obtained separately at the Index of Genomes from The CpBase: Chloroplast Genome Database (http://chloroplast.ocean.washington.edu/).

### Prediction of conserved editing sites

The Predictive RNA Editor for Plants suite (PREP-Cp) (http://prep.unl.edu/) (Mower, 2009) was used to predict conserved plastid editing sites. These sites were used to evaluate read coverage and editing percentage using the sRNA data. Fasta files corresponding to plastid coding sequence data were manually formatted to be usedfor use as an input batch file in the PREP-Cp tool. To predict editing sites for each species, a less stringent cutoff value of 0.5 was used, despite the 0.8 default value. This lower cutoff value was used to evaluate the effective occurrence of the predicted editing sites and their efficacious detection from sRNA data.

### RNA mapping and confirmation of predicted sites

**Figure 1:**
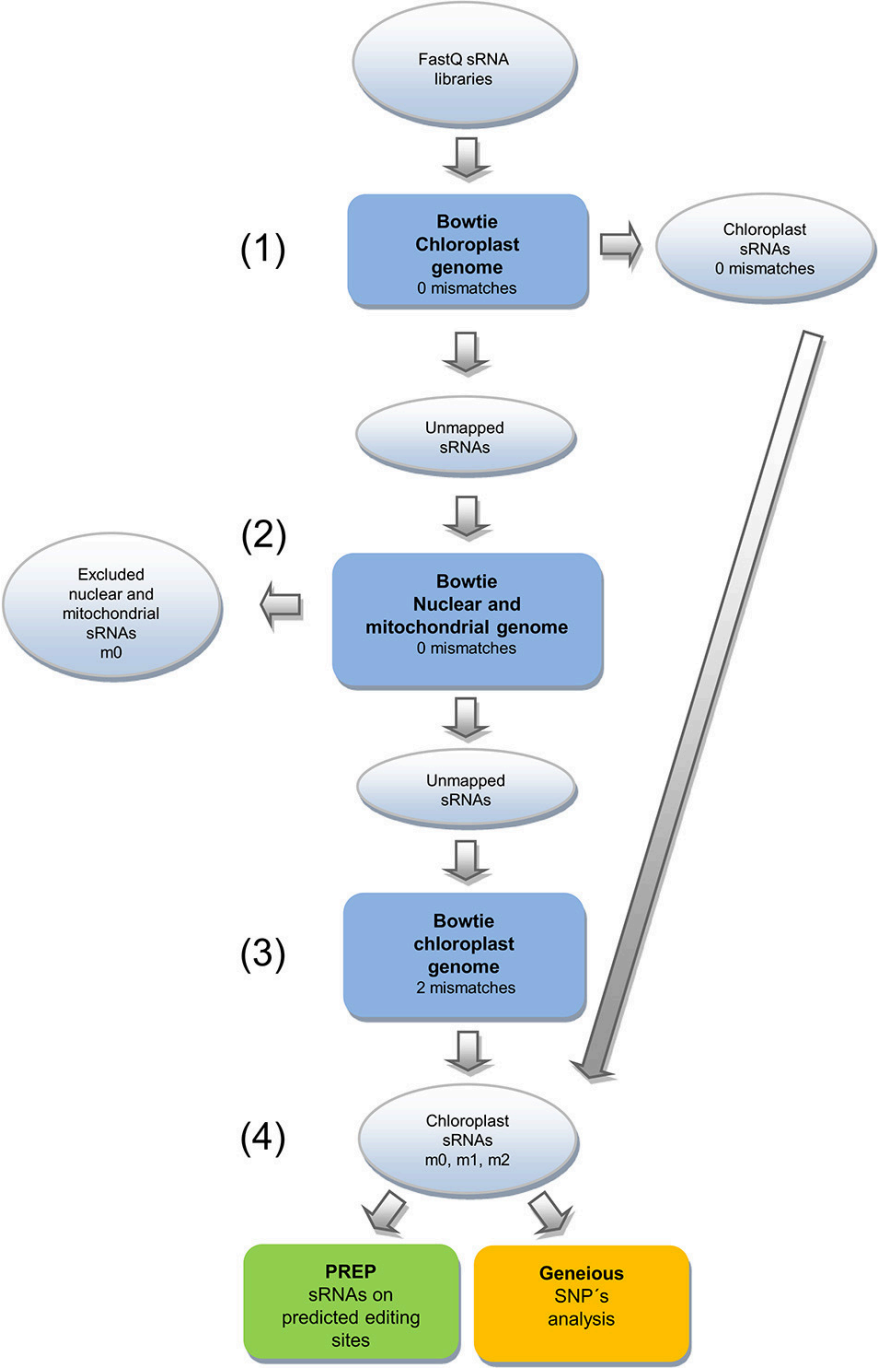
Pipeline for identification of editing sites using chloroplast RNA transcripts. (1) sRNA-seq/mRNA-seq reads were filtered by mapping against the chloroplast reference genome. Mapped reads were saved as another file named as m0 (chloroplast RNAs m0). (2) Reads that did not map were subjected to a new round of mapping against nuclear and mitochondrial reference genomes, and those reads that did map were discarded. (3) The remaining unmapped reads were remapped against the chloroplast genome allowing up to 2 mismatches using Bowtie. (4) The resulting mapped reads (chloroplast m0 + m2), plus the m0 file, were used in the analysis to predict transcript editing sites through PREP and Geneious SNPs approaches.

The sRNA/mRNA libraries were primarily mapped using Bowtie (Langmead et al., 2009) with 0 mismatch and no reverse complement against the chloroplast genome, coding sequences and tRNAs. Mapped reads resulted in a new file (m0). Unmapped reads were submitted to a second round of mapping with no mismatches against nuclear and mitochondrial genomes. This step eliminates all reads with perfect matches against these genomes. Unmapped reads were further mapped with two mismatches and no reverse complement against chloroplast genome and coding sequences. This second group of mapped reads produced another file containing reads with editing events (m2). Both m0 and m2 fastq files were concatenated in an m0 + m2 file. The C-to-U editing sites predicted by PREP-Cp in the cpDNA coding sequence were subjected to m0 + m2 mapping and further manual inspection using Tablet software (Milne et al., 2013). The predicted editing sites were confirmed based on a C-to-T mapping change. The steps described above are summarized in Figure 1.

### Single nucleotide polymorphism analysis

The m0 + m2 fastq files from sRNA libraries were mapped against the whole chloroplast genome, coding sequences and tRNAs using Geneious-R8 (Kearse et al., 2012), with the Bowtie algorithm and the same parameters of the previous mapping (Figure 1). The Geneious find variation/SNPs tool was used to search for A-to-G and C-to-T changes in putative new editing sites that were not predicted by PREP. The following parameters were used: Minimum Coverage of 5, Maximum Variant *P*-value of 10^−2^, option to find polymorphism Inside and Outside coding sequence and *P*-value calculation method as approximate. In the manual inspection of mapping, reads with putative editing events in the 5′ and 3′ end were discarded to improve prediction and selection for validation using RT-qPCR assay.

### Validation and analysis of the RNA editing sites using RT-qPCR

To validate predicted and new C-to-U RNA editing sites from the sRNA data in soybean chloroplast transcripts [*Glycine max* (L.) Merrill], we collected the roots, leaves and petals from the soybean cultivar Conquista. These tissues were collected as biological triplicates. All samples were immediately frozen in liquid nitrogen, and total RNA was extracted using Trizol (Invitrogen, CA, USA). The RNA quality was evaluated through electrophoresis on a 1% agarose gel, and the RNA amount was verified using a Qubit fluorometer and Quant-iT RNA assay kit according to the manufacturer's instructions (Invitrogen, CA, USA).

Reverse transcription quantitative polymerase chain reaction (RT-qPCR) was performed to validate the C-to-U RNA editing rates for some predicted editing sites in soybean chloroplast genes across three different tissues (roots, leaves and petals). To validate and quantify new RNA editing sites, only leaf samples were used. The cDNA synthesis was performed with approximately 1 μg of total RNA. Each reaction was primed with 1 μM dT25V oligonucleotide (Invitrogen, Carlsbad, CA, USA). Prior to transcription, RNA and the oligo(dT)25V primer oligo were mixed with RNase-free water to a total volume of 10 μL and incubated at 70°C for 5 min, followed by cooling on ice. The reactions were reverse transcribed with 1X M-MLV RT buffer, 0.5 mM dNTPs (Ludwig, Porto Alegre, RS, Brazil) and 200 U of M-MLV RT Enzyme (Promega, Madison, WI, USA) in a final volume of 30 μL. The synthesis was performed at 40°C for 60 min. All cDNA samples were diluted 100-fold with RNase-free water and subsequently used as templates in RT-qPCR analysis. The subsequent PCR amplification was performed using a set of primers designed according to Chen et al. (2008), with modifications. A set of primers, comprising two specific editing primers and one unique universal primer, were designed for each editing site. Specific editing primers were characterized by a unique difference in the last nucleotide at the 3′ end that recognizes and differentiates edited and unedited sites. All primers employed in the reaction are listed in Table S1.

All RT-qPCR reactions were performed on a Bio-Rad CFX384 real-time PCR detection system (Bio-Rad, Hercules, CA, USA) using SYBR Green I (Invitrogen, Carlsbad, CA, USA) to detect double-stranded cDNA synthesis. The reactions were conducted in a 10 μL volume containing 5 μL of diluted cDNA (1:100), 0.2X SYBR Green I, 0.1 mM dNTP, 1X PCR buffer, 3 mM MgCl_2_, 0.25 U Platinum Taq DNA Polymerase (Invitrogen, Carlsbad, CA, USA) and 200 nM of each forward and reverse primer. The samples were analyzed as biological triplicates and technical quadruplicates in a 384-well plate. A non-template control was also included. The PCR reactions were run under the following conditions: an initial polymerase hot start at 94°C for 5 min, followed by 40 cycles at 94°C for 15 s, 60°C for 15 s and 72°C for 10 s. A melting curve analysis was programmed at the end of the PCR run over the range of 65 to 99°C, and the temperature increased stepwise by 0.5°C. The threshold and baseline were manually determined using Bio-Rad CFX manager software.

To calculate the RNA editing rates, we used the threshold cycle (Ct) generated during the qPCR amplifications. To calculate the percentage of editing, an equation that considered the difference between the Ct-values of each editing variant was used:

$$\begin{array}{l} \%\;RNA\;editing\;=\\ \;\frac{2^{\left(Ct\;mean\;of\;T\;variant\;-\;Ct\;mean\;of\;C\;variant\right)}}{2^{\left(Ct\;mean\;of\;T\;variant\;-\;Ct\;mean\;of\;C\;variant\right)}+1}\;\times 100 \end{array}$$

## Results

### sRNA reads mapped to chloroplast genomes

The sRNA libraries sequenced without plastid RNA isolation were mapped to Arabidopsis, soybean and rice chloroplast genomes using an in-house pipeline (Figure 1). Approximately 3.2, 1.6, and 0.9 million reads did not map to nuclear and mitochondrial genomes but mapped to Arabidopsis, soybean and rice chloroplast genomes, respectively. These chloroplast (cp)-mapped reads represented approximately 22.9% (Arabidopsis), 4.79% (soybean), and 3.62% (rice) of the total reads in these libraries (Table 1). The editing informative m2 reads corresponded to 455,904 (Arabidopsis), 208,417 (soybean), and 144,609 (rice). The histograms representing the percentage length distribution of each individual class are shown in Figure S1. The mean coverage was 838.6 in Arabidopsis, 358.6 in soybean and 222 in rice. The maximum coverage values were 872,674 in Arabidopsis, 380,116 in soybean and 166,534 in rice. Some chloroplast regions were not covered by the sRNA library reads, with minimal coverage of zero. The number of plastid genome positions with no coverage was 47,057 in Arabidopsis, 24,505 in soybean and 3,039 in rice, representing approximately 30.46, 16.09, and 2.25% of each chloroplast genome, respectively. The genome fraction coverage for Arabidopsis, soybean and rice is represented in Figure S2.

**Table 1:** Distribution of sRNA sequences among nuclear, mitochondrial and plastid genomes.

**Organism**	**Total**	**Nuclear**	**mtDNA**	**cpDNA (m0)**	**cpDNA (m2)**	**cpDNA total**	**Not aligned**
Arabidopsis	14,113,280	6,369,985	18,393	2,778,067	454,904	3,232,971	4,491,931
	100%	45.13%	0.13%	19.68%	3.22%	22.9%	31.82%
Soybean	34,313,559	28,219,467	46,399	1,438,193	208,417	1,646,610	4,401,083
	100%	82.23%	0.13%	4.19%	0.60%	4.79%	12.82%
Rice	25,247,958	21,479,400	12,003	768,437	144,609	913,046	2,843,509
	100%	85.07%	0.05%	3.04%	0.57%	3.62%	11.27%

*m0, reads with no mismatches*.

*m2, reads with until 2 mismatches*.

### sRNA polymorphisms confirm PREP editing site prediction in coding-sequence genes

**Figure 2:**
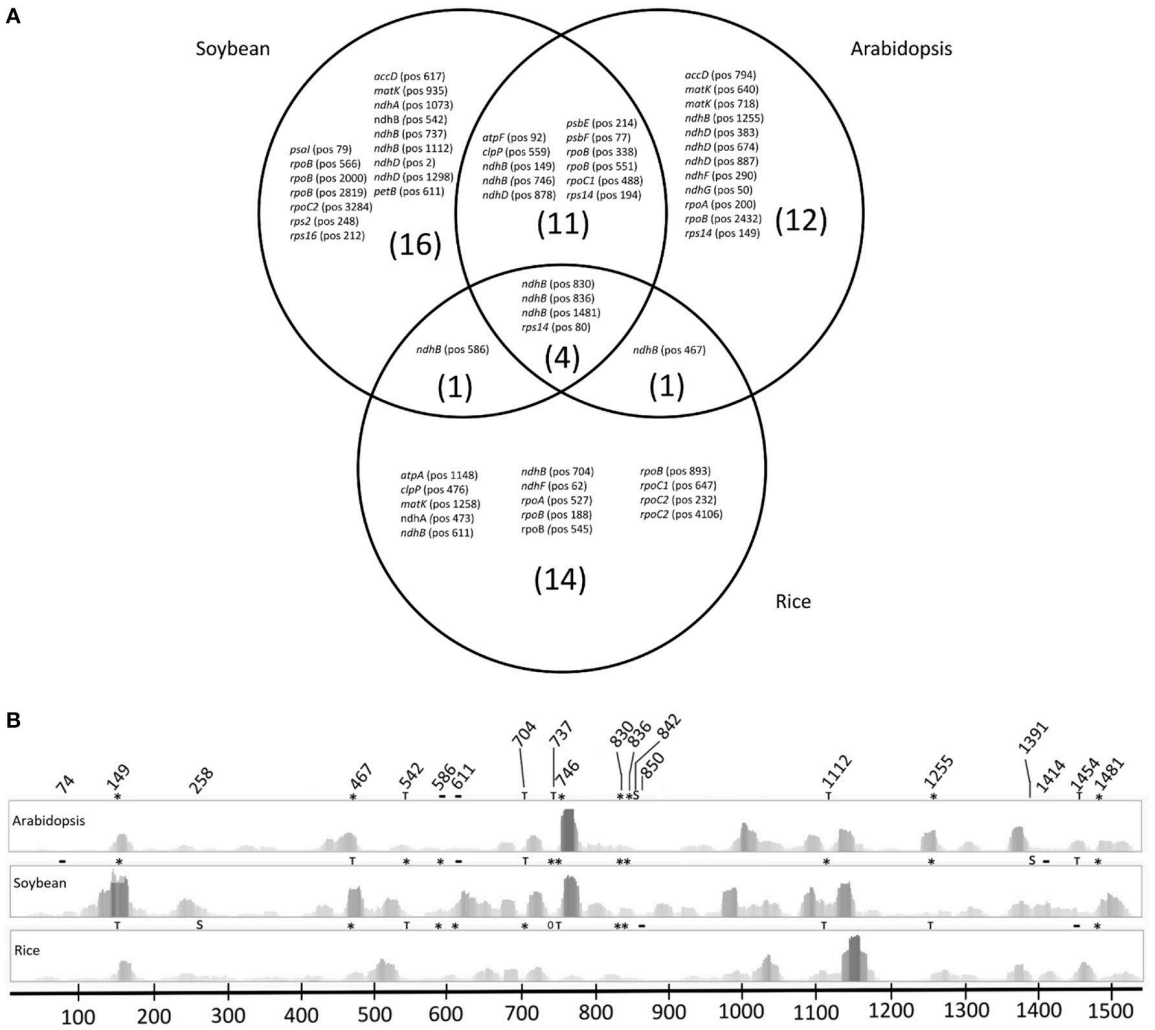
PREP predicted editing sites and graphical read distribution and editing in the *ndhB* transcript. **(A)** Venn diagram with confirmed RNA editing sites predicted by PREP in Arabidopsis, soybean and rice. Gene names followed by the position numbering of the editing site in the coding sequence are indicated. **(B)** Graphical representation of sRNA coverage and predicted editing sites in the *ndhB* gene; (S) editing sites identified by SNP analysis, (T) predicted editing site in another species that already has a thymine in the species, (^*^) editing site predicted by PREP and confirmed by read mapping and coverage, (−) predicted sites with reads but not confirmed by editing and (0) predicted editing sites without read coverage.

The conserved chloroplast C-to-U RNA editing sites were predicted using the Predictive RNA Editor for Plants (PREP-Cp) (http://prep.unl.edu/) (Mower, 2009). The PREP suite predicted 69 potential editing sites in Arabidopsis, 92 sites in soybean and 79 sites in rice chloroplast genes. These predicted editing sites were distributed in 21 different coding sequences in Arabidopsis and rice and 23 coding sequences in soybean. The mapped chloroplast sRNA reads were analyzed using Tablet software to evaluate the presence/absence of C-to-U editing events in the predicted sites. Different numbers of confirmed editing sites were observed among the three species: 28 sites in Arabidopsis, 32 sites in soybean and 20 sites in rice, corresponding to 40.57, 34.78, and 25.31% of the total sites, respectively. The PREP score (values between 0 and 1) indicates editing site prediction confidence to control the relative proportion of false positive and false negative predictions. When a more stringent score value (≥0.8) was considered, the predicted editing site numbers decreased to 45, 59, and 29 for Arabidopsis, soybean and rice, respectively. Analyses of chloroplast sRNA alignment confirmed the 23 predicted editing sites in Arabidopsis, 28 sites in soybean, and 14 sites in rice, corresponding to 51.1, 47.45, and 48.27% of the total predicted editing sites, respectively (Figure 2A). Even with a higher score value, some predicted sites were not confirmed, reflecting the absence of reads corresponding to editing or not enough coverage (Table S2). Four editing sites were conservatively predicted and confirmed among the three species. These sites corresponded to three sites inside the *ndhB* transcript and one site in the *rps14* transcript. Soybean and Arabidopsis shared 11 common editing sites in the *atpF, clpP, ndhB, ndhD, psbE, psbF, rpoB, rpoC1*, and *rps14* transcripts. Concerning the rice *atpF, clpP, ndhB, psbE*, and *psbF* genes, a thymine was already present in these editing sites. Rice shared a single editing site with Arabidopsis in the *ndhB* transcript at position 467, which in soybean corresponds to a thymine. The numbers of unique confirmed editing sites for each species were 12, 16, and 14 for Arabidopsis, soybean and rice, respectively (Figure 2A). The complete distribution of PREP predicted editing sites according to species is described in Table S2.

### mRNA-Seq and sRNA-Seq differences in RNA editing analysis

**Figure 3:**
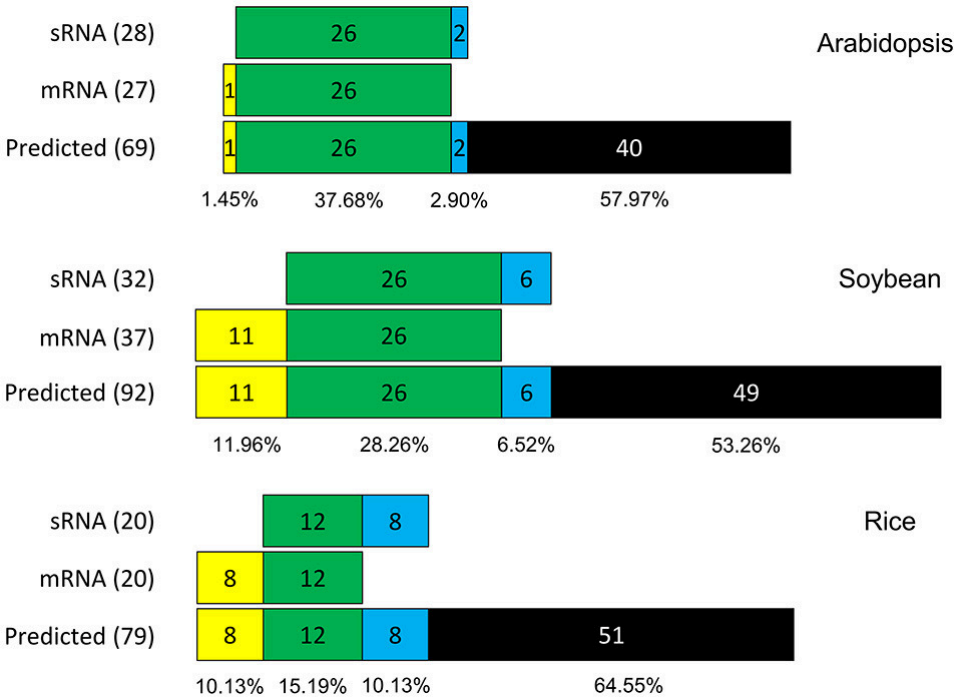
Comparison of predicted editing site confirmation between sRNA and mRNA data. On the left, values of total confirmed predicted editing sites by data type (mRNA or sRNA). Green boxes represent editing sites confirmed in both data; yellow boxes represent editing sites confirmed only in mRNA data; blue boxes represent editing sites confirmed only in sRNA data; and black boxes represent unconfirmed predicted editing sites.

To provide information concerning sRNA data reliability, the C-to-U RNA editing profiles were compared to the PREP predicted editing sites between the sRNA and mRNA (messenger RNA) libraries in Arabidopsis, soybean and rice. The mRNA-Seq data confirmed 27 predicted editing sites in Arabidopsis, 37 sites in soybean and 20 sites in rice, corresponding to 39.13, 40.21, and 25.31% of the predicted sites, respectively (Table S3). One predicted editing site was exclusively confirmed using mRNA-Seq libraries in Arabidopsis, and 11 predicted editing sites were confirmed in soybean and rice. However, analyses using sRNA-Seq libraries detected two exclusively confirmed editing sites in Arabidopsis, six sites in soybean and eight sites in rice. The confirmed predicted editing sites shared between mRNA and sRNA data corresponded to 37.68, 28.26, and 15.19% of the total predicted editing sites in Arabidopsis, soybean and rice, respectively (Figure 3).

### Confirmation of PREP predicted editing sites and new editing site prediction through SNP analysis in coding-sequences using sRNA data

In addition to the confirmation of the predicted editing sites, new candidates for editing sites were searched. A SNP analysis was used with a minimum *P*-value of ≤ 10^−10^ to identify sites with C-to-T changes. This parameter enabled the identification of 59 potential editing sites in Arabidopsis, 43 sites in soybean, and 19 sites in rice. Among these editing sites, 58, 37, and 15 sites encode amino acid changes in Arabidopsis, soybean and rice, respectively (Table S4). These editing sites were distributed in 27 genes in Arabidopsis, 24 genes in soybean and 11 genes in rice. Comparison of these editing sites against the editing sites predicted using PREP revealed that 20, 18, and 7 sites were previously predicted in Arabidopsis, soybean and rice, respectively (Table S5). Among these sites, 18, 18, and 6 sites were predicted with a higher score value in Arabidopsis, soybean and rice, respectively.

**Figure 4:**
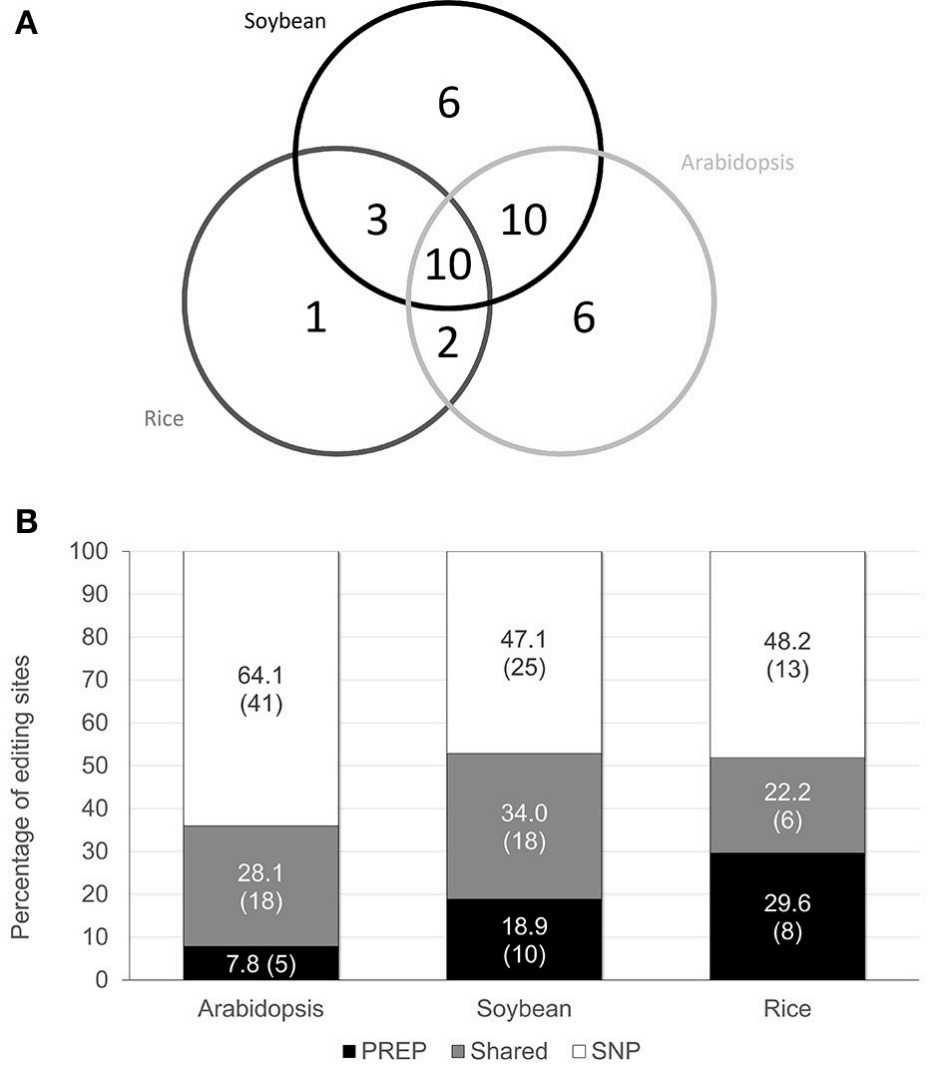
Number of genes with C-to-U editing sites in the studied species. **(A)** Venn diagram with the total number of genes with editing sites in Arabidopsis, soybean and rice, when using both PREP (only confirmed) and SNP analysis. Not all genes share common editing sites among species. The gene identities are described in Table S6. **(B)** Percentages of total RNA editing sites identified by distinct approaches, as observed in Arabidopsis, soybean and rice. The absolute number of editing sites for each method is in parentheses. Black bars correspond to the percentage of total sites confirmed only by PREP prediction (>0.8 in prediction score); white bars indicate the percentage of total sites confirmed by the SNP approach; and gray bars show the percentage of total sites confirmed using both approaches.

When the edited transcript distribution was evaluated in all species (Figure 4A), a higher editing frequency was associated with a core of genes (*clpP, ndhB, ndhF, rpoA, rpoB, rpoC1, rpoC2*, and *rps14*) and confirmed with at least one method used for all species evaluated. Considering exclusive edited genes, Arabidopsis showed 14 editing sites distributed among nine genes identified using SNP analysis. The editing in the rice *atpA* gene, detected through SNP analysis, was predicted by PREP. Soybean presented four exclusive editing sites confirmed by sRNA reads and predicted by PREP. They sites were distributed among the *petB, rps2*, and *rps14* genes. C-to-U changes promote a serine to leucine amino acid change in *petB* and *rps14* and a histidine to tyrosine amino acid change in *rps2*. Arabidopsis, soybean and rice SNP analysis revealed 19, 15, and 7 C-to-T changes distributed among 11, 10 and five exclusive genes, respectively. All genes and their respective editing sites are listed in Table S6. The comparative C-to-T analysis using different identification methods demonstrated that the SNP method could identify reliable C-to-U editing events, including events previously predicted using PREP at a lower PREP score (> 0.5) (Figure S3) or a more stringent cutoff (PREP score >0.8) (Figure 4B).

### C-to-U RNA editing in the ndhB gene

The well-studied *ndhB* gene was the most frequently edited gene detected through PREP prediction in all plants. The number of editing sites predicted by PREP in this gene varied between species: 9 sites in Arabidopsis, 13 in soybean and 10 in rice. The number of editing sites confirmed by sRNA alignment was 7 sites in Arabidopsis, 9 sites in soybean and 7 sites in rice, representing 77.7, 69.23, and 70% of the predicted editing sites, respectively. Other editing sites could not be confirmed, reflecting insufficient read coverage (Table 2). In contrast, despite high predicted editing site numbers, 7 sites in Arabidopsis, 9 sites in soybean and 5 sites in rice, the *matK* gene had only two confirmed predicted editing sites in Arabidopsis and one confirmed predicted editing site in soybean and rice (Table S2).

**Table 2:** *NdhB* C-to-U editing events by PREP and SNP approach using reads derived from sRNA-seq.

**Organism**	**Codon change**	**Nucleotide position**	**AA change**	**AA position**	**Total coverage**	**Edited coverage**	**% Editing**	**SNP *P*-value**	**PREP score**
Arabidopsis	TCA–TTA	149	S–L	50	40	32	80	4.8E-108	1
(1,539: 870)^*^	CCA–CTA	467	P–L	156	40	28	75	8.5E-109	1
	CAT–TAT	586	H–Y	196	1	0	no editing	-	1
	TCA–TTA	611	S–L	204	5	0	no editing	-	0.8
	TCT–TTT	746	S–F	249	12	5	41.7	5.3E-109	1
	TCA–TTA	830	S–L	277	20	9	45	8.4E-29	1
	TCA–TTA	836	S–L	279	21	10	47.6	1.1E-23	1
	GCC–GTC	842	T–I	281	19	2	10.5	1.3E-8	nd
	CAT–TAT	1,255	H–Y	419	47	47	100	nd	1
	CCA–CTA	1,481	P–L	494	34	14	41.2	5.5E-40	1
Soybean	CCT–CTT	74	P–L	25	4	0	no editing	nd	1
(1,533: 543)^*^	TCA–TTA	149	S–L	50	35	10	28	3.3E-11	1
	ACG–ATG	542	T–M	181	1	1	100	nd	1
	CAT–TAT	586	H–Y	196	11	2	18.2	0.0000038	1
	TCA–TTA	611	S–L	204	14	0	no editing	nd	0.8
	CCA–CTA	737	P–L	246	2	2	100	nd	1
	TCT–TTT	746	S–F	249	12	4	33.3	2.0E-14	1
	TCA–TTA	830	S–L	277	12	5	41.7	3E-17	1
	TCA–TTA	836	S–L	279	11	5	45.5	2.6E-15	1
	TCA–TTA	1,112	S–L	371	22	5	22.7	4.3E-17	1
	CAT–TAT	1,255	H–Y	419	1	0	no editing	nd	1
	CCT–CTT	1,391	P–L	464	9	2	22.7	0.0000036	
	CCC–TCC	1,414	P–S	472	10	0	no editing	nd	1
	CCA–CTA	1,481	P–L	494	13	8	64.3	1.3E-31	1
Rice	AGC–AGT	258	S–S	86	8	2	25	1.6E-8	nd
(1,533: 619)^*^	CCA–CTA	467	P–L	156	14	9	64.3	1.30E-31	1
	CAT–TAT	586	H–Y	196	5	3	60	4.00E-12	1
	TCA–TTA	611	S–L	204	2	1	50	nd	0.8
	TCC–TTC	704	S–F	235	16	3	18.8	7.10E-08	1
	CCA–CTA	737	P–L	246	0	0	nd	nd	1
	TCA–TTA	830	S–L	277	3	1	33	nd	1
	TCA–TTA	836	S–L	279	4	1	25	nd	1
	CTC–TTC	850	L–F	284	2	0	no editing	nd	0.6
	ACT–ATT	1,454	T–I	485	30	0	no editing	nd	0.6
	CCA–CTA	1,481	P–L	494	6	5	83	8.0E-17	1

* *Coding sequence length and coverage values*.

*“Nucleotide position”: position in base pair is from the A of the initiator codon*.

*“Total Coverage”: total mapped reads in respective nucleotide position*.

*“Edited Coverage”: number of reads shown T, instead C*.

*“% Editing”: percentage of RNA editing using the edited reads divided by total mapped reads*.

*“PREP score”: confidence value of prediction according PREP*.

*“nd”: no defined*.

In the *ndhB* gene, SNP analysis detected potential new editing sites in all three species (Table 2). However, this gene was not the most edited gene according to SNP analysis in rice. In this species, *ndhB* had three new potential editing sites, while *rpoC2* gene had four new sites. In Arabidopsis, *ndhD* had 8 new potential editing sites according to SNP analysis. In soybean, the *ndhB* gene remained as the most edited gene (Table S6). Comparative analyses showed a different read distribution of the predicted sites in *ndhB* among species (Figure 2B). Some regions showed higher coverage, not only in the editing site, but also in neighboring sites. For example, PREP predicted 467 editing sites (C-to-U), with varied coverage between species, but reads confirming the editing event were observed in both Arabidopsis and rice. Although soybean had a higher amount of reads in this site, a T was present in this genomic position. Notably, several sites showed more than 10 reads of coverage but did not confirm editing events. Some putative editing sites predicted using SNP analysis showed higher coverage than the predicted sites confirmed using PREP (Table 2).

### A-to-I editing events predicted using SNP analysis in chloroplast tRNA genes

Chloroplast sRNAs can also be useful in adenosine to inosine (A-to-I) RNA editing screening. tRNA genes were used to evaluate editing events, by searching for a guanosine (G) SNP in sRNA mapping since inosine is read as G by cellular machineries (Kim, 2004).

tRNA genes showed at least one position with an A-to-G change in at least two species (Table S7), totaling 11, 4, and 12 putative A-to-I editing events in Arabidopsis, soybean and rice, respectively. These A-to-G changes were distributed in 8, 4, and 10 tRNAs in Arabidopsis, soybean and rice, respectively. Among these sites, two sites were conserved between species: position 58 of tRNA-Trp (CCA) between soybean and rice and position 35 of tRNA-Arg (ACG) among all species evaluated. In tRNA-Arg (ACG), nucleotide 35 presented 40, 58.8, and 67.8% of the edited reads in Arabidopsis, soybean and rice, respectively (Table 3). The tRNAs most frequently edited were tRNA-Ser (UGA), with 3 A-to-G changes in Arabidopsis, and tRNA-Leu (UAG) and tRNA-Trp (CCA) with two A-to-G changes in Arabidopsis and rice, respectively.

**Table 3:** A-to-I editing analysis of tRNA-Arg(ACG) sites by SNP approach with corresponding reads derived from sRNA-seq.

**Organism**	**Nucleotide position**	**Nucleotide change**	**Total coverage**	**Edited coverage**	**% Editing**	**Variant *P*-value**
Arabidopsis	35	A–G	80	32	40	3.8E-655
(74: 3,015)^*^						
Soybean	35	A–G	80	47	58.5	2.3E-144
(74: 65,787)^*^						
Rice	35	A–G	214	145	67.8	1.5E-465
(74: 1,673)^*^						

* *tRNA sequence length and coverage values*.

*“Total Coverage”: total mapped reads in respective nucleotide position*.

*“Edited Coverage”: number of reads shown G, instead A*.

*“% Editing”: percentage of RNA editing using the edited reads divided by total mapped reads*.

### Validation of C-to-U RNA editing in soybean plastid genes

**Figure 5:**
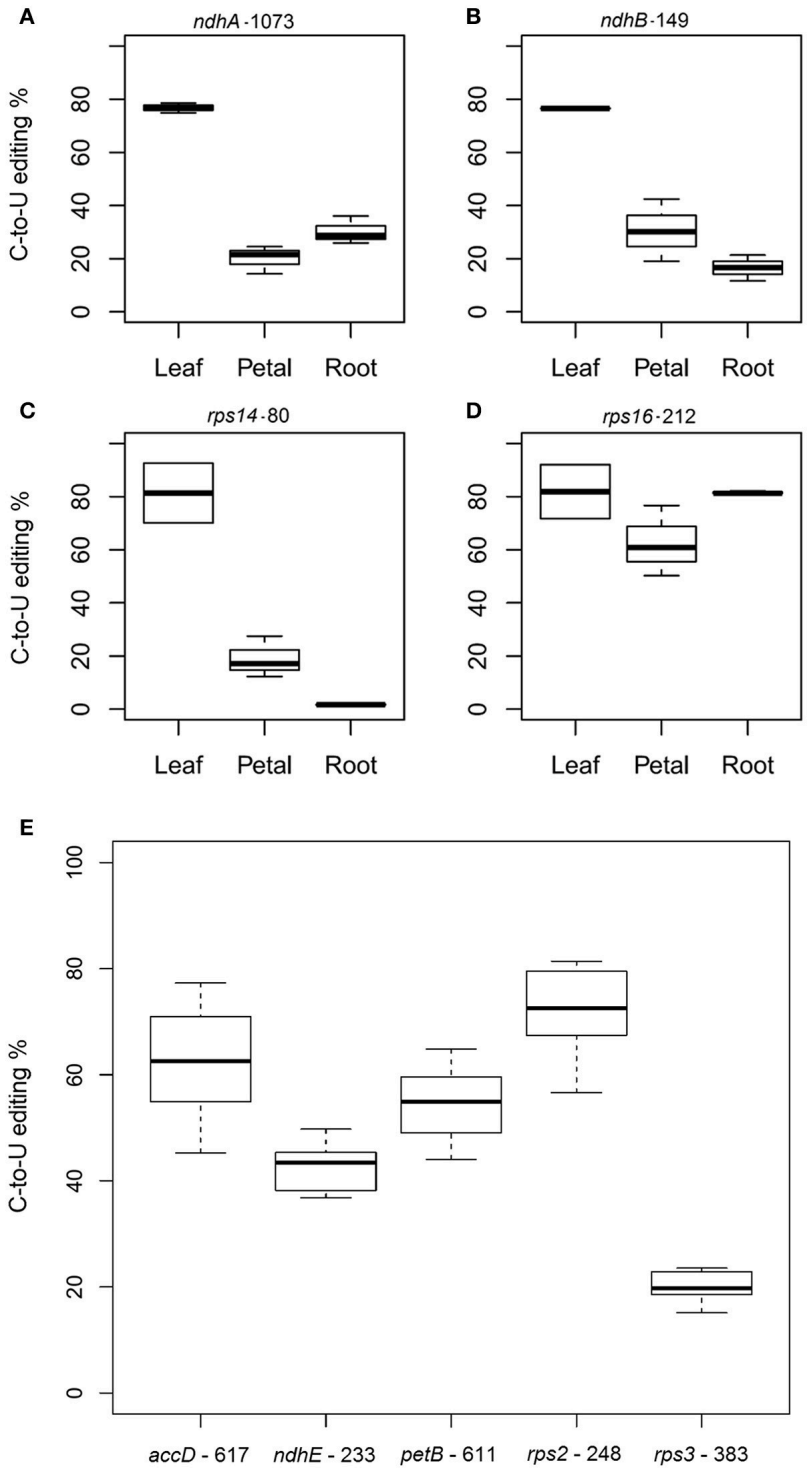
Confirmation and quantitation of soybean editing sites predicted by PREP. **(A)** *ndhA-1053*, **(B)** *ndhB*-149, **(C)** *rps14*-80, and **(D)** *rps16*-212 were analyzed in leaves, petals and roots. Box area represents the lower and upper percentiles; **(E)** confirmation and quantitation of soybean editing sites identified by SNP analysis. Transcripts from soybean leaves were analyzed for C-to-U editing in specific nucleotide positions: *accD*-617, *ndhE*-233, *petB*-611, *rps2*-248, and *rps3*-383. Box area represents the lower and upper percentiles. The upper whisker of the boxplot indicates the highest editing value observed; the lower whisker, the lowest editing value; and the middle line, the median.

To validate some predicted editing sites and demonstrate sRNA data reliability as a resourceful tool for the identification of RNA editing sites, four PREP predicted editing sites were selected for C-to-U RNA editing analysis using RT-qPCR. The *ndhA* (position 1073), *ndhB* (position 149), *rps14* (position 80), and *rps16* (position 212) editing sites were comparatively quantified in different soybean tissues (Figures 5A–D). Five new putative editing sites, identified by SNP analysis, were also confirmed and quantified in leaf samples: *accD* (position 617), *ndhE* (position 233), *petB* (position 611), *rps2* (position 248), and *rps3* (position 383) (Figure 5E). RT-qPCR showed that the percentage of *ndhA* editing was higher in leaves (76.75%) than in petals (20.11%) or roots (30.23%) (Figure 5A). The same editing pattern was observed for *ndhB* and *rps14*. In *ndhB*, the percentage editing was 72.41, 30.54, and 16.55% (Figure 5B), while values of 74, 17.86, and 8.15% were obtained in *rps14* editing in the leaves, petals and roots, respectively (Figure 5C). The *rps16* editing profile was different, with an editing percentage that was higher than 60% in all tissues (Figure 5D). With respect to putative new C-to-U editing sites identified using SNP analysis, RT-qPCR confirmed C-to-U editing events and demonstrated different editing rates among genes: *accD* (60.2%), *ndhE* (39.85%,) *petB* (54.3%), *rps2* (71.52%), and *rps3* (20.02%) (Figure 5E).

## Discussion

In the present study, we propose an additional resource and new method to identify conserved and new RNA editing sites in plastid RNA sequences. Currently, an increasing number of high-throughput sequencing data have become available. Among these datasets, there are substantial data corresponding to sRNA sequencing libraries. After analyzing some of these libraries, we observed that even without previous isolation of chloroplasts for further RNA extraction and sequencing, millions of chloroplast-derived sRNA reads could be recovered, reflecting mapping against the chloroplast genome. An important constraint of the presented method refers to the library quality and the read coverage of reference genomes.

In the present study, Arabidopsis libraries had the highest mean coverage using sRNA reads, which likely facilitated the recovery of the largest number of confirmed editing sites. The coverage percentage across genomes was different between species, with lower values detected in Arabidopsis. This result demonstrated that the use of sRNA libraries for mapping editing events is not directly related to a significant coverage across the entire plastid genome. Although this method has the capacity to confirm and discover editing sites in chloroplasts, a smaller number of mitochondrial reads would likely affect RNA editing analysis in this organelle. In the present study, the approach for the identification of editing sites was compared to the PREP and SNP strategies. The editing sites and percentage editing may vary between species because some species may already possess a thymine in the genome. In these cases, C-to-U editing will not occur. The same situation can occur with some A-to-I editing sites, which could affect the general percentage of editing among species. The use of a different PREP score, resulting in distinct cut-off values, may also affect these percentages. In addition, editing factors and their editing sites may evolve differently among species.

The elementary step employed in the pipeline used in the present study was the initial sRNA library mapping against the chloroplast genome, considering 0 mismatches. Plastid DNA insertions in nuclear genomes have been demonstrated for partial, intact or even truncated coding sequences in several species (Chen et al., 2015). Thus, an initial filtration step against the chloroplast genome prevents the loss of unedited reads to those loci present in nuclear insertions. Unedited reads are necessary, particularly in quantitative editing analysis, where the editing percentage is measured and cannot be ruled out.

Some C-to-U editing studies have previously used mRNA-Seq to demonstrate and quantify editing events in plant mitochondria (Bentolila et al., 2013) and chloroplasts (Guo et al., 2015). Comparison of sRNAs and mRNA data sequences demonstrated that most of the confirmed editing sites can be recovered using both datasets. However, there are differences between these data, demonstrating that sRNAs can identify editing sites that were not detected using mRNA data and vice versa (Figure 3). The use of sRNA data to complement RNA editing analysis can improve the identification and measurement of RNA editing in various aspects.

In the present study, a new set of plastid editing sites was identified in soybean. The C-to-U editing events have previously been demonstrated in other species, and we recovered several edited transcripts, including *ndhB, ndhD, ndhG, rpoB*, and *rpoC1* (Corneille et al., 2000; Okuda et al., 2009; Zhou et al., 2009; Chateigner-Boutin et al., 2011; Boussardon et al., 2012; Tseng et al., 2013), in the present analysis. For most known C-to-U editing sites predicted through PREP and confirmed by sRNA reads in the present study, 21 sites have previously been demonstrated in Arabidopsis (Tsudzuki et al., 2001; Tillich et al., 2005) and 19 sites have previously been demonstrated in rice (Corneille et al., 2000; Tsudzuki et al., 2001), representing 30.43 and 24% of the total predicted editing sites, respectively (Table S2). Moreover, we showed editing events in soybean plastid genes, including *ndhA, psaI*, and *petB*, which had not previously been demonstrated for rice or Arabidopsis. In the SNP analysis, we identified new C-to-U editing sites. For example, in the Arabidopsis *ndhF* gene, a putative C-to-U editing site was identified at position 884, leading to a serine to phenylalanine change. In the soybean *ndhE* gene, a putative C-to-U editing site at position 233 was observed in 73.7% of the reads. This editing led to a proline to leucine change in the encoded protein. Despite this information, the impact of amino acid modifications on respective protein structures remains unclear. Both *ndh* genes encode thylakoid Ndh complex components involved in photosynthesis optimization under different stress conditions conditions (Casano, 2001; Martin et al., 2004; Rumeau et al., 2007). *NdhB* mutants under lower air humidity conditions or following exposure to ABA present a reduction in the photosynthetic level, likely mediated through stomatal closure triggered under these conditions (Horvath, 2000). Therefore, a protein structure modification, resulting from a loss or decrease in RNA editing events could affect adaptations to stress conditions or cause other unknown changes.

The coding sequence of protein D2, encoded by the *psbD* gene, a photosystem II (PSII) core protein, showed a putative new editing event in rice at positions 1006 and 1007. However, reflecting low coverage, these new editing sites still require further experimental confirmation. Maintenance of the D2 protein structure is important not only for proton transport (Pokhrel et al., 2013) but also for the phosphorylation dynamics of this protein (Tikkanen and Aro, 2012) and its interaction with the proteins responsible for PSII maintenance (Liu and Last, 2015). If this editing site is confirmed, then alterations in editing site patterns resulting from factors, such as abiotic stress could be associated with photo-oxidative damage susceptibility. Previous studies have demonstrated that abiotic stress influences the editing process and consequently plastid physiology (Nakajima and Mulligan, 2001; Karcher and Bock, 2002).

Five putative C-to-U editing sites predicted using SNP analysis were validated through RT-qPCR. This result demonstrates the reliability and accuracy of sRNA data resources and the method presented herein to confirm predicted sites *in silico* and identify new RNA editing sites. Position 1073 in the *ndhA* gene is an editing site identified only in the soybean chloroplast editome. RT-qPCR revealed that the editing percentage varies among different soybean tissues. The *ndhB* (position 149) gene was previously evaluated in the non-photosynthetic tissues of Arabidopsis. An RNA editing pattern previously demonstrated in Arabidopsis (Tseng et al., 2013), with a higher percentage in leaves (>75% edited), followed by flowers (25–75% edited) and roots (unedited), was similarly observed in the present study. An exception was observed for the root tissue, which showed a low editing percentage (16.5%) in soybean instead of an unedited rate, as observed in Arabidopsis. The editing site at position 80 in *rps14* also was evaluated across different tissues in Arabidopsis. A high editing percentage was demonstrated in Arabidopsis leaves (Tseng et al., 2013), a pattern also demonstrated in soybean using RT-qPCR. The RNA editing percentages observed in roots and petals showed different patterns between Arabidopsis and soybean, although a decrease in these values was observed in the root tissue of both species. The editing of *rps16* at position 212 was predicted and confirmed only in soybean and did not show differences in the editing percentage between leaf and root tissues. These results indicate that sRNA sequence mapping can not only be used to confirm the predicted editing sites, but also to quantify the editing percentage.

The plastid acetyl-CoA carboxylase, necessary for *de novo* fatty acid synthesis, comprises two components, *accA* and *accD* proteins; *accD* encodes the β-carboxyl transferase subunit and is required in tobacco plants for a functional enzyme (Kode et al., 2005). The vanilla cream1 (vac1) albino mutant, reflecting a PPR-DYW protein required for editing in *accD* and *ndhF* in Arabidopsis, exhibits albino to pale yellow phenotype and an RNA editing reduction in those transcripts (Tseng et al., 2010). The requirement of plastid *accD* editing for functional protein has previously been demonstrated (Sasaki et al., 2001), and this new editing site, which promotes a serine to leucine change, could also be important for the maintenance of protein structure and functionality. The *ndhE* gene encodes a subunit of a membrane subcomplex of the NAD(P)H dehydrogenase complex (Peng et al., 2011). NdhE protein interacts with the membrane subcomplex proteins, NdhC and NdhG, and with subcomplex proteins, NhdH and NdhK (Efremov et al., 2010; Peng et al., 2011). The new editing site described here promotes a proline to leucine change, which could modify the interaction between these proteins and lead to changes in electron transfer to quinone. The *petB* gene encodes the cytochrome *b*_6_ protein, a cytochrome *b*_6_*f* complex component responsible for mediating electron transfer between photosystem I (PSI) and plastocyanin (Baniulis et al., 2008); mutants of *petB* in tobacco showed reduced levels of PSI, PSII and light-harvesting complex proteins (Monde et al., 2000), indicating a requirement of cytochrome *b*_6_ to correct photosynthetic apparatus assembly. The new editing site involving a serine to leucine change in *petB* at position 611, identified in the present study, could be required for the maintenance of cytochrome *b*_6_*f* complex structure and stability. Proteins S2 and S3 are located on the solvent side of ribosome small subunit (Manuell et al., 2004), and RNA editing events can modify their interactions among other ribosomal proteins and likely with mRNA, with potential effects on the regulatory aspects of plastid translation in response to stress or other homeostasis processes.

The SNP analysis facilitated the evaluation of not only C-to-U editing but also A-to-I editing events in chloroplast tRNAs. The tRNA-Arg (ACG) A-to-I editing event was also observed in all three species in the present study. This change corresponds to an inosine in the wobble position, which encodes three arginine codons CGU, CGC, and CGA that play a critical role in plastid protein synthesis (Rogalski et al., 2008). The enzyme involved in this mechanism in Arabidopsis, At1g68720, encodes a tRNA adenosine deaminase (TADA), which is targeted to plastids. RNAi lines of this gene show markedly reduced A-to-I editing efficiency, displaying phenotype consequences, such as growth and development delays (Elias and Huang, 2005; Delannoy et al., 2009; Karcher and Bock, 2009). Editing events in others tRNAs have been shown in some species and have been well studied in animals (Su and Randau, 2011) and previously demonstrated in moss *Takakia lepidozioides* (Miyata et al., 2008). The method described here can help to identify and measure other tRNA editing events not yet described in plants.

In addition to the high amount of data currently available in public databases that can readily be assessed, there are some plastid sRNAs biological features that can reveal important mechanisms of RNA editing. The precise plastid sRNA biogenesis remains unknown because there is no evidence of any RNAi machinery in organelles that could originate small RNAs thus far. Notably, there is evidence of a relaxed plastid genome transcription mechanism, resulting in full plastid genome transcription (Hotto et al., 2012). It has been suggested that plastid sRNAs originated from RNA sequence regions protected against degradation by forming secondary structures or from associations with RNA-binding proteins regions (Pfalz et al., 2009). The results of the present study demonstrated that sRNAs are not necessarily over-represented in regions of editing sites but are also evident in coding sequences with smaller lengths, where these sRNAs can still be observed. These biological features enable the use of sRNA datasets to confirm the results of different RNA editing prediction tools and enable the analysis of editing events not only in a qualitative but also a quantitative manner, depending on the library quality and read coverage.

The identification of editing sites and measurement of editing levels have demonstrated differences among tissues (Tseng et al., 2013) and developmental stages (Miyata and Sugita, 2004). These findings can be used to evaluate the impact of different stresses on these mechanisms (Nakajima and Mulligan, 2001; Van Den Bekerom et al., 2013). Thus, the use of sRNA data to confirm predicted editing sites in association with SNP searches can provide a powerful and reliable plastid editome characterization and measurement, and the results can be applied to compare editing levels in different tissues, developmental stages and physiological conditions.

## Conclusion

Analysis of sRNA libraries can be used to identify and quantify RNA editing events. Using this source of sequence data and pipeline of analyses, we obtained, for the first time, a consistent set of non-conserved and new editing sites in soybean. We propose the use of plastid sRNA libraries as a novel source and approach to study RNA editing events. Until recently, no other studies have taken advantage of such data to screen for RNA editing sites. Thus, the results from the present study should encourage researchers to use small RNA libraries to compare RNA editing in different plants under different conditions to improve knowledge on the editing role of plastid RNA in plant biology.

## Author contributions

RM, NR, and AC conceived and designed the study. NR conducted *in silico* analysis. NR and FK conducted the RT-qPCR experiments. NR and GdF analyzed the data. NR and AC drafted the manuscript. All authors have read and approved the manuscript.

### Conflict of interest statement

The authors declare that the research was conducted in the absence of any commercial or financial relationships that could be construed as a potential conflict of interest.

## Supplementary material

Figure S1 sRNA length distribution. The histograms represent the percentage of length distribution of each individual class. In black, gray and white bars, Arabidopsis, soybean, and rice read data, respectively.

Figure S2 Number of plastid genomic sites (Y-axis) and their respective sRNA reads coverage (X-axis). In black, gray and white bars, Arabidopsis, soybean and rice read data, respectively.

Figure S3 RNA editing site numbers identified by the PREP and SNP approaches in Arabidopsis, soybean and rice. Black bars correspond to sites confirmed only by PREP prediction (>0.5 in prediction score); white bars indicate sites confirmed using the SNP approach; and gray bars show sites confirmed using both approaches.
