# Supplementary figures and images for: Unveiling Chloroplast RNA Editing Events Using Next Generation Small RNA Sequencing Data
Source: Front Plant Sci. 2017 Sep 29;8:1686. doi: 10.3389/fpls.2017.01686 (PMC5626879; doi:10.3389/fpls.2017.01686)

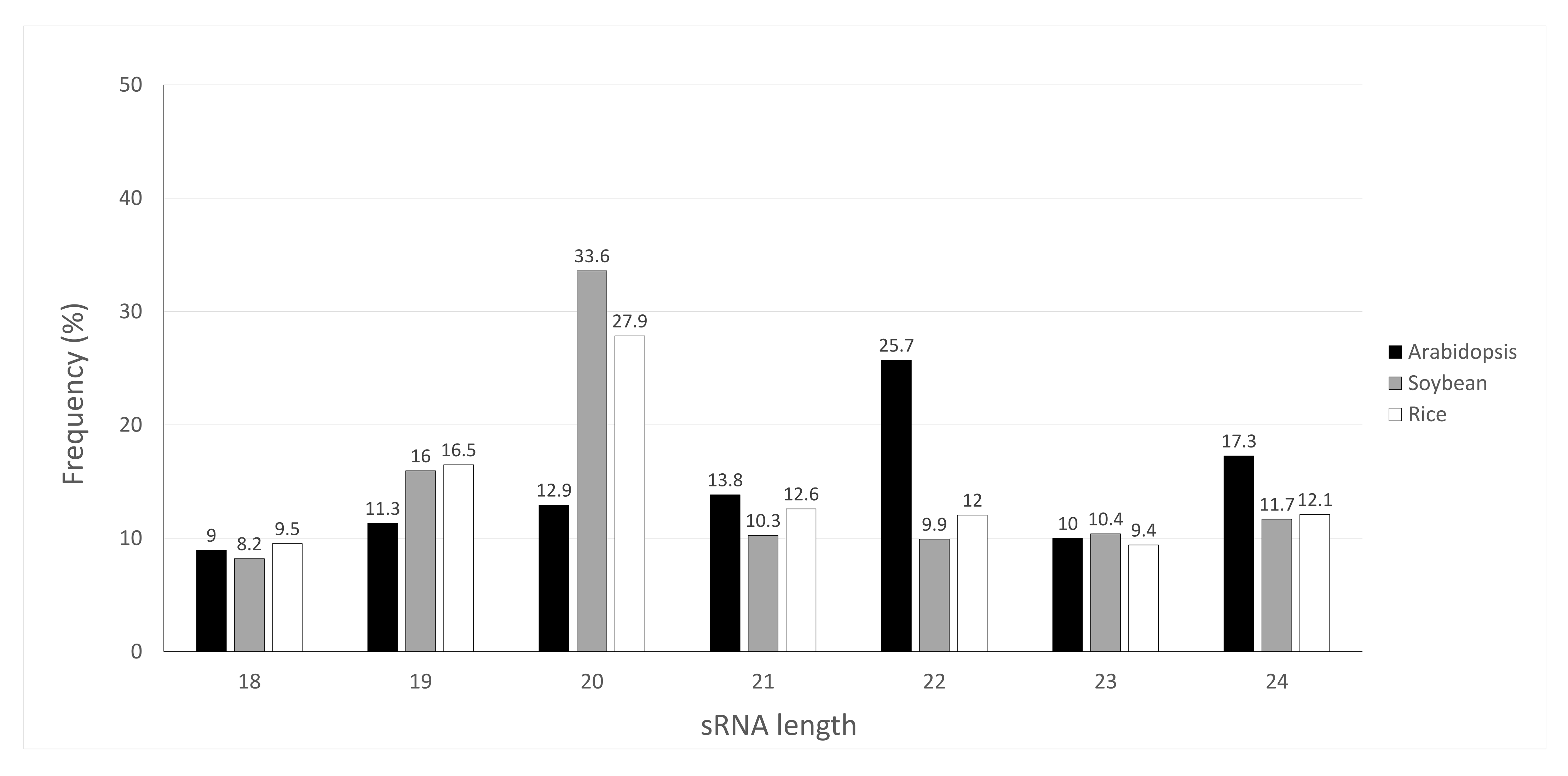

Supplement: Figure S1 — sRNA length distribution. The histograms represent the percentage of length distribution of each individual class. In black, gray and white bars, Arabidopsis, soybean, and rice read data, respectively. [file Image1.TIFF]

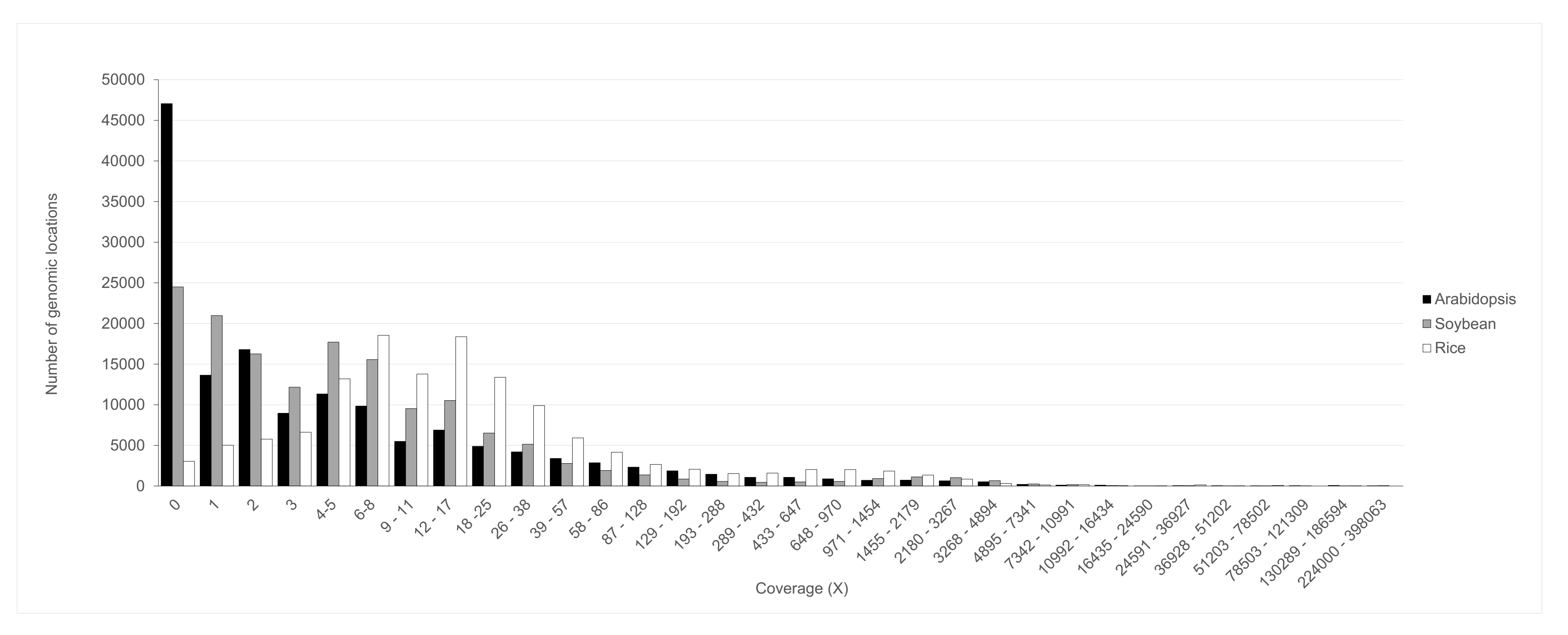

Supplement: Figure S2 — Number of plastid genomic sites (Y-axis) and their respective sRNA reads coverage (X-axis). In black, gray and white bars, Arabidopsis, soybean and rice read data, respectively. [file Image2.TIFF]

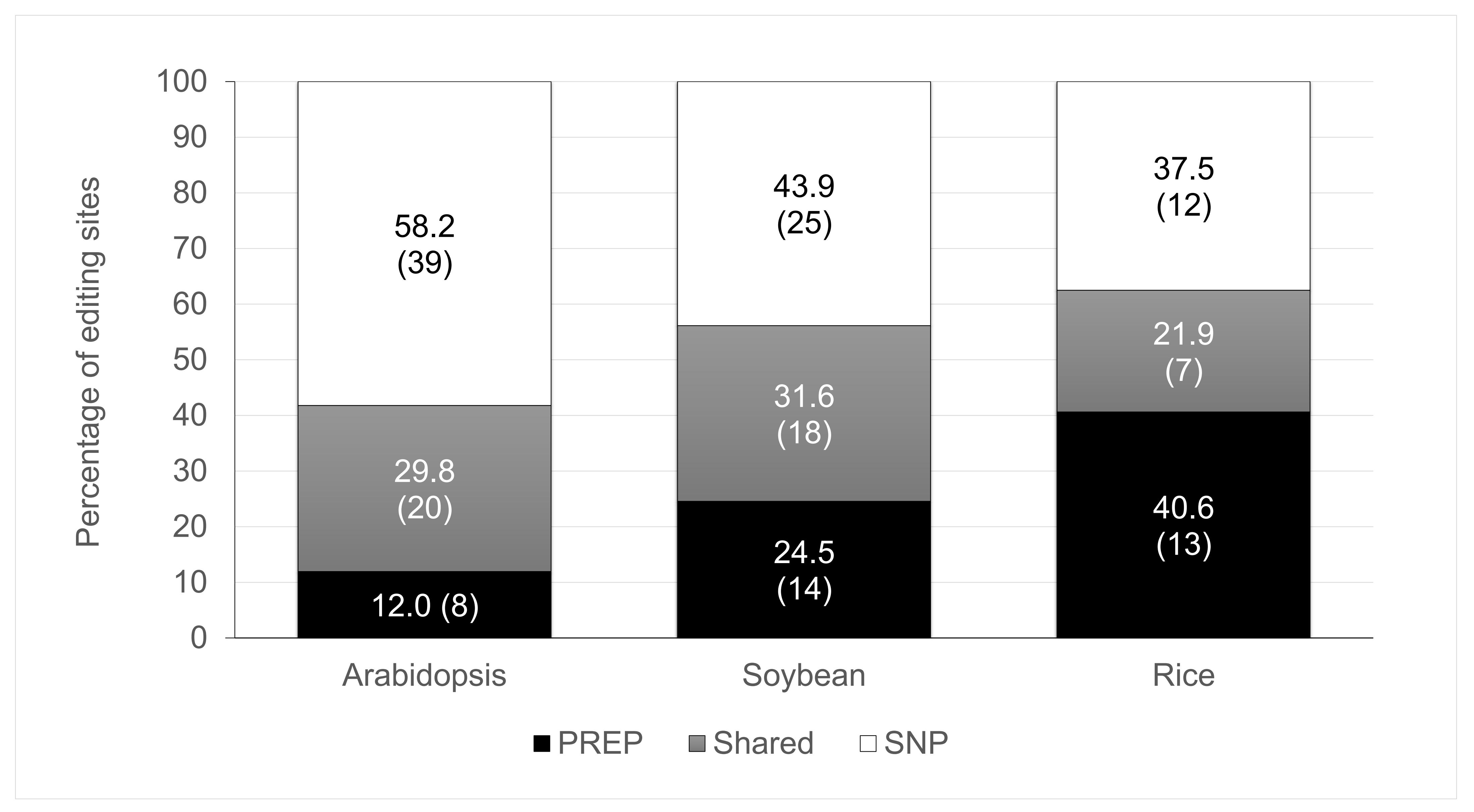

Supplement: Figure S3 — RNA editing site numbers identified by the PREP and SNP approaches in Arabidopsis, soybean and rice. Black bars correspond to sites confirmed only by PREP prediction (>0.5 in prediction score); white bars indicate sites confirmed using the SNP approach; and gray bars show sites confirmed using both approaches. [file Image3.TIFF]
